# Association Between Venous Mesenteric Thrombosis and Plasminogen Activator Inhibitor-1 Mutation

**DOI:** 10.7759/cureus.75974

**Published:** 2024-12-18

**Authors:** Vânia Pereira, Daniel Castanheira, Marta Sanches, Beatriz Castro Silva, Raquel Almeida

**Affiliations:** 1 Internal Medicine Department, Hospital Beatriz Ângelo, ULS Loures Odivelas, Loures, PRT

**Keywords:** acute mesenteric ischemia (ami), direct-acting oral anticoagulants, plasminogen activator inhibitor-1 (pai-1), superior mesenteric vein thrombosis, thrombophilia screen

## Abstract

Plasminogen activator inhibitor-1 (PAI-1) is central to fibrinolysis regulation, and genetic variants such as the 4G/4G genotype predispose individuals to hypercoagulability. This case highlights a 46-year-old female patient presenting with acute mesenteric venous thrombosis, where genetic evaluation revealed homozygosity for the PAI-1 4G/4G polymorphism. Management with unfractionated heparin followed by a transition to direct oral anticoagulants led to clinical resolution. This report underscores the role of PAI-1 polymorphisms in thrombotic pathophysiology and long-term anticoagulation therapy.

## Introduction

Mesenteric venous thrombosis (MVT) is a life-threatening condition due to its association with mesenteric ischemia, carrying a mortality rate of 12 and 20%. It accounts for 5-15% of all cases of mesenteric ischemia. A multitude of risk factors have been identified, including liver cirrhosis, malignancy, coagulation disorders, intra-abdominal infection/inflammation, and postoperative states. Diagnosing acute MVT based solely on clinical findings is challenging due to its variable presentation. While some patients remain asymptomatic with MVT incidentally detected during imaging, others experience severe symptoms such as abdominal pain, gastrointestinal bleeding, vomiting, and diarrhea. In extreme cases, patients may present with acute abdomen and signs of sepsis. Computerized tomography (CT) with angiography is the diagnostic tool of choice. Presentation with acute abdomen necessitates immediate surgical intervention. Additionally, the administration of unfractionated heparin upon diagnosis is critical to prevent extension of the thrombus. Early diagnostic efforts should prioritize identifying local disease processes and coagulation disorders, as these have significant therapeutic implications [1,2].

Thrombophilia is defined as an abnormal predisposition to form a thrombus and can result from inherited mutations, acquired conditions, or more commonly, an interaction between both. PAI-1, a serine proteinase inhibitor, plays a major role in the endogenous fibrinolytic system by inhibiting tissue plasminogen activators, thereby reducing the generation of active plasmin. Overexpression of PAI-1 may result in the impairment of the fibrinolytic system and further increase the risk of thrombotic events. The guanine insertion/deletion polymorphism (4G/5G) within the PAI-1 promoter gene can impact the expression of PAI-1. The 5G/5G polymorphism is considered a normal variant in the population and may present as heterozygosity (5G/4G) or homozygosity (4G/4G or 5G/5G). The 4G allele is slightly more transcriptionally active, and people with homozygosity for the 4G allele showed increased plasma PAI-1 concentrations compared to people with a 5H allele, heightening their risk of venous thromboembolism (VTE) [3-7].

We present a case of a patient with mesenteric venous thrombosis and homozygosity for the 4G/4G PAI-1 genotype.

## Case presentation

A 46-year-old female patient, with a past medical history of fibromyalgia and using a hormonal oral contraceptive, presented to the Emergency Department with complaints of early satiety and epigastric discomfort lasting for four days, which progressed to generalized abdominal pain accompanied by bilious and alimentary emesis, without hematocheazia, on the day of presentation. 

On physical examination, her abdomen exhibited preserved bowel sounds, but was diffusely tender to both superficial and deep palpation, with maximal tenderness in the left flank and iliac fossa. Initial laboratory workup demonstrated leukocytosis with marked neutrophilia and an elevated C-reactive protein. Renal function was unremarkable and liver enzymes were within normal limits (Table 1).

**Table 1:** Laboratory findings on admission INR: International Normalized Ratio, PT: prothrombin time, aPTT: activated partial thromboplastin time, PCO_2_: partial pressure of carbon dioxide, HCO_3_: bicarbonate

Parameters (unit)	Patient values	Reference values
Hemoglobin (g/dL)	12.3	12-15
Leukocytes (10^9^/L)	15.57	4.5-11
Neutrophils (%)	84.5	40-70
Platelets (10⁹/L)	224	150-450
INR	1.1	0.8-1.11
PT (seconds)	13.1	9.4-12.5
aPTT (seconds)	21.8	25.1-36.5
Fibrinogen (g/L)	5.5	2-4
Total bilirubin (mg/dL)	0.32	<0.9
Conjugated bilirubin (mg/dL)	0.16	<0.5
Aspartate aminotransferase (U/L)	16	<32
Alanine amilotransfererase (U/L)	18	<33
Alkaline phosphatase (U/L)	61	35-104
Gamma-glutamyl transferase (U/L)	18	<40
Lactate dehydrogenase (U/L)	210	135-214
Creatine kinase (U/L)	48	<170
Creatinine (mg/dL)	0.85	0.51-0.95
Urea (mg/dL)	39	16.6-48.5
Sodium (mmol/L)	138	136-145
Potassium (mmol/L)	4	3.5-5.10
Chloride (mmol/L)	101	98-107
C-reactive protein (mg/L)	135	<5
pH	7.45	7.35-7.45
pCO_2 _(mmol/L)	30	35-48
HCO_3_ (mmol/L)	20.9	21-28
Lactate (mmol/L)	1.1	<1.2

**Figure 1:**
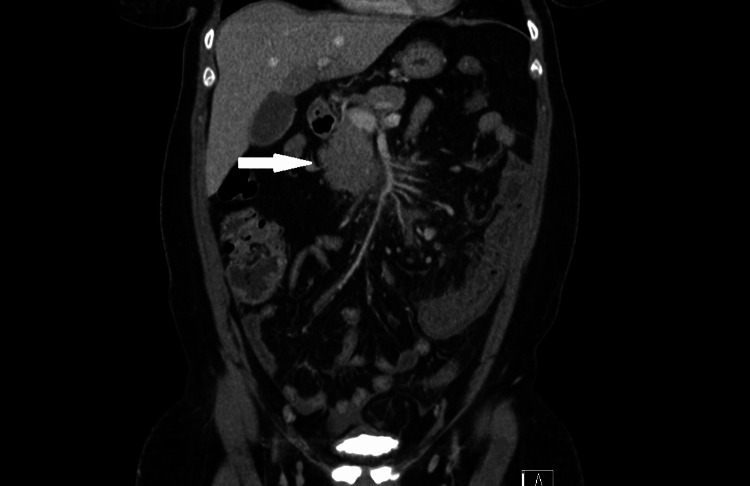
Coronal CT imaging of superior mesenteric vein thrombosis Coronal CT angiography scan highlighting a thrombus in the superior mesenteric vein (arrow)

CT angiogram of the abdomen identified extensive acute thrombosis of the superior mesenteric vein (SMV), almost completely occluding its lumen. Associated findings included significant congestive densification of the mesenteric root, moderate ascites, multiple regional lymphadenopathies, and stratified mural thickening of several small bowel loops, consistent with venous ischemia (Figure 1). 

In light of the substantial risk for intestinal ischemia, the case was discussed with general surgery and interventional radiology teams. Given the patient’s clinical stability and controlled pain, analgesic, surgical intervention, or endovascular therapy was not urgently indicated. Anticoagulation with unfractionated heparin was initiated promptly.

A comprehensive evaluation for venous thromboembolic risk factors was performed. Cross-sectional imaging and endoscopic studies excluded an underlying malignancy, while gynecological evaluation ruled out reproductive tract and breast pathology. Thrombophilia testing revealed homozygosity for the plasminogen activator inhibitor-1 (PAI-1) 4G/4G mutation, which is known to confer a prothrombotic phenotype. The remainder of the thrombophilia panel, including antiphospholipid antibodies, was unremarkable (Table 2).

**Table 2:** Laboratory assesment for thrombophilia screening IgG: immunoglobulin G, IgM: immunoglobulin M, JAK 2: Janus kinase 2, MTHFR: methylenetetrahydrofolate reductase, PAI1: plasminogen activator inhibitor 1

Parameters (unit)	Patient values	Reference values
Lupus anticoagulant	Negative	-
Anti-cardiolipin antibodies IgG	3.2	<20
Anti-cardiolipin antibodies IgM	<1	<20
Anti-beta2 glycoprotein antibodies IgG	<6.4	<20
Anti-beta2 glycoprotein antibodies IgM	<1.1	<20
Protein C activity (%)	121	70-140
Protein S activity (%)	90	60-140
Antithrombin activity (%)	86	83-128
Prothrombin 20210A mutation	Negative	-
JAK 2 mutation	Negative	-
Factor V Leiden mutation	Negative	-
MTHFR C667T e 1298C mutation	Negative	-
PAI-1 mutation	Mutation 4G/4G	-

The patient’s clinical course was favorable, with progressive resolution of abdominal pain and restoration of oral intake. By the fifth day of hospitalization, anticoagulation was transitioned to low molecular weight heparin (LMWH). Repeat imaging demonstrated a significant reduction in thrombus burden within the SMV, although not yet fully patent, with recanalization of several tributary branches and decreased inflammatory changes. Upon discharge, anticoagulation therapy was transitioned to apixaban at a dose of 5 mg twice daily. The hormonal contraceptive method was also discontinued due to being an additional risk factor.

## Discussion

Impaired fibrinolysis, driven in part by overexpression of PAI-1, plays a significant role in thrombus formation and hemostatic imbalance. Although several studies associate the 4G allele with an increased predisposition to thrombosis, particularly in internal organs’ blood vessels, the overall evidence remains controversial [4,7].

Associated conditions such as factor V Leiden mutations and myeloproliferative disorders, are identified in up to 55% of MVT cases. Routine thrombophilia testing in mesenteric thrombosis is debatable; although it may influence patient treatment and risk stratification, its cost, potential for misinterpretation, and impact on the patient's quality of life remain negative factors [8]. Testing is especially recommended in patients without another identifiable cause. Indefinite anticoagulation is commonly recommended for patients with significant pro-thrombotic factors, despite limited evidence for its impact on long-term outcomes [2]. In the case of our patient, the PAI-1 test was conducted after more common abnormalities typically associated with this condition were ruled out. Although the patient was using hormonal contraception, which can be a risk factor for VTE, given the extent of the thrombus, it was decided to perform a thrombophilia study.

Anticoagulation therapy is the cornerstone of MVT management, aiming to prevent thrombus propagation, facilitate recanalization, and reduce the risk of recurrence, in order to prevent complications such as intestinal infarction or portal hypertension. Initial treatment involves unfractionated heparin or LMWH, followed by oral vitamin K antagonists (VKAs) or direct oral anticoagulants (DOAC). In our case, we decided to start unfractionated heparin because it has a shorter half-life, in case the patient needed emergency surgery. Although no specific trials have been performed on DOAC treatment after MVT, there is no reason to believe that these agents would not be as effective as VKA [9]. The recommended treatment duration is generally three to six months, but indefinite therapy is advised for patients with persistent pro-thrombotic conditions. Recent advancements in endovascular techniques, such as transjugular intrahepatic portosystemic shunt (TIPS) with thrombectomy or thrombolysis, percutaneous transhepatic thrombectomy or thrombolysis, and thrombolysis via the superior mesenteric artery provide options for cases refractory to anticoagulation, although evidence is limited to case series [1-2]. Some centers are also using systemic thrombolysis in these cases [10]. In cases of bowel ischemia, perforation, or peritonitis, surgical intervention is required to resect non-viable bowel while preserving as much tissue as possible [1,2].

Monitoring treatment effectiveness, typically with follow-up imaging, helps determine the necessity of prolonged anticoagulation. Complete recanalization occurs in approximately 50% of treated MVT patients and partial recanalization in 40%, with low rates of hemorrhagic complications. At the three-month follow-up consultation, our patient showed a favorable response both clinical and radiological, prompting the decision to continue long-term anticoagulation therapy based on the identified mutation [2].

Further research is required to clarify the relationship between PAI-1 polymorphisms and VTE, as well as their broader implications for clinical practice.

## Conclusions

This case underscores the importance of prompt recognition and management of acute mesenteric vein thrombosis. Early anticoagulation, multidisciplinary coordination, and vigilant monitoring were pivotal in achieving a favorable outcome without the need for invasive interventions. The complexity of MVT underscores the need for individualized treatment strategies, balancing anticoagulation benefits against bleeding risks, and integrating emerging therapies where applicable.

Identification of the PAI-1 homozygous mutation as a contributory factor highlights the importance of tailored thrombophilia screening in patients presenting with atypical venous thromboembolic events. Direct oral anticoagulants are effective in managing thrombotic events in such patients, offering a practical and safe alternative to traditional therapies.
